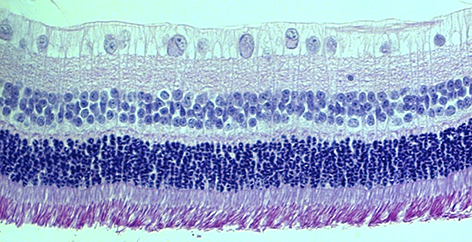# Vitamin A dimers as primary drivers of retinal degeneration

**Published:** 2015-02

Eye diseases that lead to blindness, including age-related macular degeneration and Stargardt disease, are characterised by the accumulation of vitamin A dimers (such as A2E) in the retinal pigment epithelium (RPE) and Bruch’s membrane (BM; an extracellular matrix located between the retina and choroid). Although vitamin A dimers are important physiological players in the visual cycle, their role in retinal degeneration is still poorly understood. To elucidate this, Ilyas Washington and colleagues investigated the effects of intravenous administration of A2E, obtained by a single-step chromatography-free method, in rabbits. The authors found that A2E induced photoreceptor damage together with inflammation and remodelling of blood vessels in the RPE and the supporting BM, leading to retinal degeneration. These results suggest that accumulation of vitamin A dimers is not a secondary symptom but rather a primary driver of retinal degeneration. Targeting vitamin A dimerisation might represent a strategy to prevent several forms of blindness. **Page 131**

**Figure:**